# Stress and Learning in Pupils: Neuroscience Evidence and its Relevance for Teachers

**DOI:** 10.1111/mbe.12282

**Published:** 2021-02-28

**Authors:** Sue B. Whiting, Sam V. Wass, Simon Green, Michael S. C. Thomas

**Affiliations:** ^1^ Department of Psychological Sciences Birkbeck, University of London, London, UK; ^2^ School of Psychology University of East London, London, UK; ^3^ Centre for Educational Neuroscience Birkbeck, University of London, London, UK

## Abstract

Our understanding of how stress affects primary school children's attention and learning has developed rapidly. We know that children experience differing levels of stressors (factors that cause stress) in their environments, and that this can influence how they respond to new stressors when they occur in educational contexts. Here, we review evidence showing that stress can increase children's attention and learning capacities in some circumstances but hinder them in others. We show how children differ in their attention and learning styles, dependent on stress levels: for example, more highly stressed children may be more distracted by superficial features and may find it harder to engage in planning and voluntary control. We review intervention research on stress management techniques in children, concentrating on psychological techniques (such as mindfulness and stress reappraisal), physiological techniques (such as breathing exercises) and environmental factors (such as reducing noise). At the current time, raising teachers' awareness of pupils' differing stress responses will be an important step in accommodating the differing needs of children in their classrooms.

## Abstract 2

**Lay Abstract**: Our understanding of how stress affects primary school children's attention and learning has developed rapidly. In this review, we consider how children's different experience of stress in their environment can influence how they respond to further stress experienced at school; we show how stress can increase attention and learning in some circumstances but hinder them in others; and how children differ in their attention and learning styles depending on stress levels. Finally, we consider the effectiveness of interventions intended to improve children's resilience.

Almost everyone has experienced common symptoms of stress, such as a raised heart rate, excessive sweating and a dry mouth. But not everyone appreciates that these are part of the body's ‘fight or flight response’ – a system that has evolved to maximize our body's capacity to respond to actual physical danger (Cacioppo, Tassinary, & Berntson, 2000). We now know that, in addition to these bodily changes, stress also associates with other, more subtle mental changes – it influences, for example, how we decide what to pay attention to in our environment (Arnsten, 2009); and we know that children are as affected by stress – if not even more so – than are adults (Koss & Gunnar, 2018).

The amount of stress that we are exposed to has a long‐term influence on multiple aspects of our neural and physical development (Dufford, Kim, & Evans, 2020). For example, adverse life events (such as changing parental figures in the home, frequently moving house, domestic violence or abuse) can lead to long‐term effects (Felitti et al., 1998), particularly if children are exposed to them during early life (Gunnar & Quevedo, 2007; Koss & Gunnar, 2018). Evidence suggests that individuals are sensitive to environmental influences in different ways (Pluess et al., 2018), and, as some degree of resilience or vulnerability runs in families, genetic inheritance may also be involved (Keers et al., 2016; Kuijper et al., 2019).

We also know that the effects of stress exposure are detectable both immediately, and over longer timescales (Schwabe & Wolf, 2014). Because of this, a cumulative effect of stressors may occur within an individual who is already in a stressful state when encountering a subsequent stressor (Joëls, Fernandez, & Roozendaal, 2011). Finally, we are starting to understand how an individual's stress level affects how they pay attention and learn. In educational settings, an individual's level of stress response in a given situation influences their learning capacities in complex ways (Schwabe & Wolf, 2014).

Many of the initial neuroscience findings on the effects of stress that stemmed from rodent studies are now being reflected in human studies (Heim, Entringer, & Buss, 2019; Romens, McDonald, Svaren, & Pollak, 2015; Zannas & West, 2014). However, there are ethical considerations and other added complexities when dealing with humans, as opposed to groups of genetically identical rodents where it is possible to deliberately manipulate the carefully controlled laboratory conditions. Moreover, the majority of the human literature on the relationship between stress and learning stems from adult studies. Nevertheless, there is little reason to suspect that the relationships shown in adults should not also be present in children (Quas, Castro, Bryce, & Granger, 2018; Quas, Yim, Edelstein, Cahill, & Rush, 2011; Quas, Yim, Rush, & Sumaroka, 2012). In this review article, we focus where the research exists on a consideration of stress as it pertains to learning in primary age children. We argue that it is important to understand, and take account of, the effects of stress on children's learning in the education system, while acknowledging that research has yet to fully characterize this relationship.

This review is structured in four parts. In Part (a), we begin by describing what stress is and how our brains and bodies respond to stress at different timescales. In Part (b), we explain what determines how much of a stress response is elicited in a particular situation. In Part (c), we describe how an individual's stress response may influence their learning. Finally, in Part (d), we review classroom interventions that use this knowledge in different ways with the goal of using our understanding of stress to improve learning outcomes.

## (A) WHAT IS STRESS – AND HOW DOES OUR BRAIN/BODY RESPOND TO STRESS AT SHORT/LONG TIMESCALES?

The term ‘stress’ is used with a variety of different meanings by researchers in different fields (Epel et al., 2018). In psychology and neuroscience, a distinction is commonly made between short‐term (or ‘acute’) stress arising from factors that have a clear start and end point (e.g., a child being frightened by a dog in the park) and long‐term (or ‘chronic’) stress arising from factors that have no clear end or recovery phase (e.g., poverty, parental neglect) (Epel et al., 2018).

Our brains and bodies respond to different sorts of stress in a multitude of different ways. Animal research initially demonstrated how the stress response actually consists of a number of different types of response, occurring over different timescales (Joëls & Baram, 2009). Immediately on encountering something that causes stress (known as a ‘stressor’), changes are triggered in different signaling chemicals in the brain (Joëls & Baram, 2009). As a result, there is a change in activity in brain areas involved in arousal, alertness, and attention (particularly sustained attention) (Joëls et al., 2011). These changes alter the way that we pay attention and learn in complex ways (see Part (c)).

In addition, various physiological changes occur in the body, aimed at maximizing the body's capacity to respond to an anticipated physical challenge (Cacioppo et al., 2000; Ulrich‐Lai & Herman, 2009). These changes include elevated heart rate (to maximize blood supply to the muscles), sweating (to cool the body in anticipation of exertion), expansion of the bronchial tubes in the lungs, and the release of short‐term energy stores. Together, these responses are known as the fast‐acting sympathetic nervous system (‘fight or flight’) response. There are also reductions in activity in the parasympathetic nervous system, which is involved in the more slow‐acting and response‐dampening (‘rest or digest’) responses, e.g., decreased reproductive function, and decreased saliva production and digestive function (Cacioppo et al., 2000). A well‐studied marker of the parasympathetic nervous system is heart rate variability, a measure of beat‐to‐beat variations in our heart rate. High heart rate variability is associated with low stress.

More slow‐acting chemical responses are also triggered over a range of timescales from minutes to hours. The most well‐known of these is called cortisol. The changes induced by these chemicals operate using different modes of action (Vogel & Schwabe, 2016) and alter brain and bodily function in a range of different ways (Engel & Gunnar, 2020) (see Part (c)). One of these effects is that cortisol temporarily suppresses the immune system response, in order to maximize the resources available for immediate, short‐term survival, an effect initially demonstrated in animals (e.g., Westly & Kelley, 1984). This effect has been attributed as the reason why students so often report infections, such as a cold sore, during the exam season (McGregor, Murphy, Albano, & Ceballos, 2016).

Swift recovery from a fast stress response is considered to be adaptive (Del Giudice, Ellis, & Shirtcliff, 2011; McEwen & Wingfield, 2003), the threat being speedily dealt with through additional energy resources so the individual may return to normal. The process through which our body is able to achieve stability through change – consisting of active processes by which an organism responds to daily events in order to maintain its normal balance or equilibrium – is known as allostasis (McEwen & Wingfield, 2003). However, the process of disrupting and restoring equilibrium carries with it a cost. The term ‘allostatic load’ (McEwen & Stellar, 1993) refers to the long‐term ‘wear and tear’ which accumulates when an individual is exposed repeatedly to stressful situations. Long‐term effects of repeated exposure to stressors have been documented extensively throughout the brain and body (McEwen, 2017). As a result of these changes, the total amount of stress to which someone has been exposed over their lifespan also influences how they respond to new stressors when they occur (see Part (b)).

Stress may also be considered as a relationship between an individual and the environment that involves an appraisal of the stressor's significance and whether extra resources are required (Lazarus & Folkman, 1984). The appraisal is the individual's evaluative judgment of the situation or event – i.e., whether it presents a threat or a challenge. This will be shaped both by personal and environmental factors (Epel et al., 2018). Therefore, an individual's stress response is largely determined by his or her interpretation of an event, rather than the event itself (Dickerson & Kemeny, 2004). Although unpredictability and novelty are such psychological factors (Mason, 1968), stronger determinants, particularly during motivated performance tasks, are an out‐of‐control feeling and a social‐evaluative threat (e.g., negative judgment by others) (Dickerson & Kemeny, 2004).

‘Resilience’ is the ‘capacity to prepare for, recover from and adapt in the face of stress, adversity, trauma or tragedy’ (Heartmath, 2020). The word ‘capacity’ implies the possibility of increasing one's capability to deal with stress by using the analogy of an inner battery (Heartmath, 2020), and suggests not only bouncing‐back and recouping faster but also neutralizing or even preventing some of the negative consequences.

## (B) WHAT LONG‐TERM FACTORS DETERMINE AN INDIVIDUAL'S LEVEL OF STRESS RESPONDING IN A GIVEN SITUATION?

Sixty‐one percent of adults, in a survey spanning 23 USA states, reported that they had suffered an adverse childhood experience (such as physical, emotional or sexual abuse, incarcerated household member, parental separation or divorce) between the ages of 0–17 years (Merrick, Ford, Ports, & Guinn, 2018). Regrettably, considerable evidence now suggests that early‐life stress is associated with lifelong effects, including elevated risk of physical disease (Danese & McEwen, 2012), higher lifelong incidence of all types of mental health problems (Conway, Raposa, Hammen, & Brennan, 2018), and cognitive impairments in older adult humans (Lupien, Maheu, Tu, Fiocco, & Schramek, 2007).

Evidence suggests that some individuals are more sensitive than others to environmental influences, and some of these differences may be due to genetic inheritance i.e., some degree of resilience or vulnerability runs in families (Keers et al., 2016; Kuijper et al., 2019). For example, hair cortisol concentration can be used as a marker of long‐term stress response activity. One twin study involving adolescents and young adults estimated the heritability of hair cortisol concentration at 72% (Rietschel, Streit, Zhu, et al., 2017). Differences in stress response may also be transmitted between generations by environmental pathways. For example, epigenetic research has suggested that a mother experiencing stress might condition her offspring either to a metabolism that is more alert to danger, i.e., a more easily triggered stress response ready for fight or flight (Babenko, Kovalchuk, & Metz, 2015), or one with a blunted stress response, i.e., insufficient arousal (Shakiba, Ellis, Bush, & Boyce, 2019).

Perhaps surprisingly, children who are more sensitive to environmental influences have been argued to respond differently not only to adversity (e.g., poverty and maltreatment) but also to positive experiences (e.g., sensitive parenting and social support) (Boyce, 2016; Pluess et al., 2018; Wass, 2018). According the Orchid‐Dandelion model, children are either environmentally sensitive, ‘orchid’ children (Ellis & Boyce, 2008), who suffer most in adverse conditions but also benefit most from optimum conditions; or less sensitive, ‘dandelion’ children, who are able to thrive anywhere (Ellis, Boyce, Belsky, & Bakermans‐Kranenburg, 2011). These groups have been consistently noted across many samples of children, adolescents and adults, although younger children may be more environmentally sensitive than the other age groups (Pluess et al., 2018). An alternative model, Diathesis‐Stress, offers no upside of greater sensitivity, instead arguing that genetics or early life events serve to make children more vulnerable to the effects of later environmental stressors, as risk factors accumulate (e.g., Grasso, Ford, & Briggs‐Gowan, 2013; see Slagt, Dubas, Dekovic, & van Aken, 2016 for an appraisal of these competing models).

Emotional neglect during childhood has been associated with long‐term changes in parts of the brain involved in memory and learning, which appear to be most severe in the brain regions involved in processing emotional information (Bogdan, Williamson, & Hariri, 2012; Chattarji, Tomar, Suvrathan, Ghosh, & Rahman, 2015; Goodman et al., 2018; McGowan et al., 2009; Quesada, Wiemers, Schoofs, & Wolf, 2012; Romens et al., 2015; Turecki & Meaney, 2016). In addition, exposure to early life stress is thought to disrupt the body's feedback systems that help re‐establish stability after we are exposed to stress (de Kloet, Joëls, & Holsboer, 2005; Denver, 2009).

Increased long‐term stress is also most commonly associated with an increased response to new stressors when they occur, along with an inability to recover from the effects of stress exposure (Gunnar & Quevedo, 2007). However, a competing proposal is that children's exposure to *small* amounts of long‐term stress can act as a ‘stress inoculation’, improving the ability to cope with new stressors when they occur – demonstrated by rodent models and then extended to humans (Chaby et al., 2015; Parker, Buckmaster, Hyde, Schatzberg, & Lyons, 2019; Russo, Murrough, Han, Charney, & Nestler, 2012). One way to reconcile these findings is to suggest that children who show the biggest response to new stressors are those who have either the highest, or the lowest levels of life‐long stress exposure. Children who show the lowest response to new stressors are those who have experienced intermediate levels of life‐long stress (Ellis & Boyce, 2008). Alternatively, a history of successfully navigating (perhaps more modest) stress‐inducing situations may furnish individuals with a greater sense of agency, thereby reducing out‐of‐control feelings, and attenuating the cognitive appraisal component of future stress responses.

## (C) HOW DOES AN INDIVIDUAL'S LEVEL OF STRESS RESPONDING AT A GIVEN MOMENT IN TIME INFLUENCE THEIR LEARNING CAPACITIES AT THAT MOMENT?

**Fig 1:**
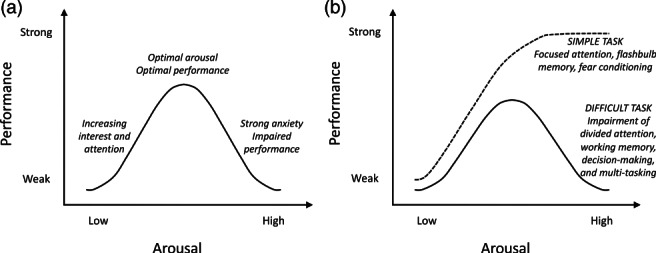
(a) The Yerkes and Dodson Law (Yerkes & Dodson, 1908). Diamond, Campbell, Park, Halonen, and Zoladz (2007) describe this version of the Law as that found in five decades of publications and books on memory, such as in Hebb (1955), Loftus (1980), and Radvansky (2006). (b) Diamond et al.'s (2007) depiction of the original version of the Law, based on the actual findings of Yerkes and Dodson on fear conditioning in mice. The original version captures the observation that strong emotionality can enhance performance under simple learning conditions. Under more difficult learning conditions, strong emotionality attenuates performance. Simple learning conditions would involve, for example, focused attention on a restricted range of cues, while complex or difficult learning conditions would involve, for example, divided attention, multi‐tasking, or greater working memory demands. Figure is adapted from Diamond et al. (2007, Figure 2).

A useful heuristic, characterizing broad patterns of responding to stress, is known as the Yerkes‐Dodson Law (see Figure 1) (Diamond et al., 2007; Yerkes & Dodson, 1908). A converging picture has emerged from animal research, research with human adults and research with developmental populations. Changes in physiological arousal affect how individuals allocate their attention due to changes in anticipatory awareness (Murphy, Robertson, Balsters, & O'Connell, 2011; Wass, 2018). According to this concept, best attention and learning is observed in children at intermediate levels between under‐aroused (dropping off to sleep) and over‐aroused (hyperactive and agitated). Using tasks where participants have to look out for a particular sought‐for object among multiple distractors, findings suggest that under‐aroused children fail to respond to the sought‐for object when it appears; by contrast, over‐aroused children respond indiscriminately, to both the sought‐for object and distractors. Only the group at intermediate levels of arousal responded to the sought‐for target, but not the distractors (Aston‐Jones & Cohen, 2005; Wass, Daubney, Golan, Logan, & Kushnerenko, 2019). Evidence consistent with this view has been shown using manipulations that induce temporary high‐stress states in adults (Arnsten, 2009), and in studies that examine naturally varying stress states in children and infants (Wass, Daubney, et al., 2019).

The term ‘arousal’ corresponds to levels of activity within the autonomic nervous system, the system responsible for control of the bodily functions that are not consciously directed, such as breathing and heart rate. Higher levels of activity within this system indicate temporarily elevated physiological stress. Individual differences in vulnerability, stress responsiveness and psychological perception of events (see above) potentially affect the shape (height, width or symmetry) of the inverted‐U function that links arousal and performance (Sapolsky, 2015). As a result, along with evidence that different children may show different levels of stress responding in a given situation (as described in Part (b)), the differing stress responses to learning tasks conceivably impact learning in a variety of different ways, and may also depend on the demands of the task.

Elevated short‐term stress is associated with immediate decreases in activity in brain regions associated with response planning and inhibition (Liston, McEwen, & Casey, 2009), along with increased activity in brain areas associated with maintaining vigilance (Arnsten, 2009). Research examining how behavior is affected by short‐term stress suggests that working memory, planning and response inhibition are impaired by temporarily elevated stress, while other cognitive functions such as rapid learning are actually improved (Arnsten, 2009). Although this research has mainly been conducted with adults, recent research has shown consistent results in children (de Barbaro, Clackson, & Wass, 2016). For example, young children being raised in more densely populated urban environments show elevated physiological stress in home settings. When tested in the laboratory, the same children showed *reduced* sustained attention but *faster* speeds of learning (Wass, Smith, Stubbs, Clackson, & Mirza, 2019). This ties in with the ‘hidden talents’ model of adaptive intelligence, in which although early‐life adversity can undermine healthy development, children growing up in harsh environments may also develop typical, or even enhanced skills for solving problems in high‐adversity contexts (the hidden talents) (Ellis et al., 2020). These talents may include increased creativity, and superior skills in switching and novel information tracking in similar stressed environments (see Section titled ‘Interventions’).

However, the fact that stress is involved with a cascade of different types of chemical response means that the effects of stress on attention and learning may differ at longer time intervals between the stress exposure and learning. For example, whereas stress during or just before learning may strengthen memory, stress experienced some time before the learning event can impair memory (see Figure 2 in Vogel & Schwabe, 2016). Vogel and Schwabe (2016) proposes that stress alters the balance of the different learning and memory systems, thus critically shaping the quality and quantity of memories (see Figure 5 in Vogel & Schwabe, 2016). A study with university students, using several measures of stress, demonstrated this effect (Zoladz et al., 2011). Positive emotional content learned at the time of the stressful event was better retained (demonstrating a significant positive correlation with heart rate during stress manipulation) but negative emotional content learned 30 minutes afterwards was more poorly retained (demonstrating a significant negative correlation with their blood pressure and salivary cortisol level changes during the stress manipulation). Zoladz and colleagues suggested that different biological mechanisms potentially contribute to differential timing effects that prior stress exerts on learning. The rationale behind these time‐dependent effects on learning and memory may be understood by taking an adaptive perspective and considering selective pressures on primitive survival mechanisms. Vogel and Schwabe speculate that the different waves of chemical changes in the brain have evolved both to improve our learning capacity at the time of stressful events – because remembering these types of events will be helpful for our future survival – and to reduce neural excitability afterward, so the body may return to normal. To appraise a potentially stressful event, existing memories would need to be quickly drawn upon to compare the current incident with previous similar ones. Future survival would be enhanced by avoidance of similar life‐threatening encounters, implying that strong memories of the event would be essential. Suppressing memories unrelated to the stressor immediately subsequent to the event would minimize memory interference, thus further strengthening the already strong memory of the relevant event (Schwabe & Wolf, 2014).

## (D) CLASSROOM INTERVENTIONS TO OPTIMISE CHILDREN'S STRESS RESPONSE

We have seen how some stress (demonstrated by an increase in children's arousal and attention) is required for successful learning; however, too much or too little stress associates with less successful learning. Children will respond to stressors differently because of certain long‐term factors (e.g., genetics, environment) and short‐term factors (e.g., recent stress exposure before arriving at school). Because of these individual differences, a positive challenge for one child may under or over‐stimulate another child, thus potentially reducing effective learning. A child's response may vary from day to day, or even during the same school day, depending on their appraisal of the situation and their available coping strategies. At high stress, children are more distracted by superficial features of the environment, will find it harder to engage in planning and voluntary control of their attention, and are more likely to engage in habitual behaviors due to reduced efficiency of executive functions such as inhibitory control.

In the discussion which follows, we focus on three types of intervention to alleviate the negative impact of stress: first, those that focus on psychological factors underlying the stress response; second, physiological interventions to reduce stress in children; third, those that focus on environmental factors. While the majority focus on stress reduction as the main goal of the intervention, some studies are concerned with the subsequent impact on test performance and attention (e.g., Khng, 2017).

### Psychological Factors

#### Reappraising Stress

The notion of psychological stress has often been perceived as too vague and wider‐ranging to measure accurately (Crosswell & Lockwood, 2020). However, there are several well‐established ways to measure stress levels, including behavioral coding, self‐report measures, and using physiological measurements such as cortisol and heart rate variability; the reader is referred to Crosswell and Lockwood (2020) for a review of the different methods. The main psychological factors behind the stress response (Dickerson & Kemeny, 2004; Mason, 1968) are unpredictability, novelty, out‐of‐control feeling and a social‐evaluative threat. Of these, the last two produce the strongest effect during motivated performance tasks (Dickerson & Kemeny, 2004; Mason, 1968). Thus, the degree of stress responding that a child shows in a given situation can be influenced by whether a child knows that he/she will be judged by his teacher and possibly others in his class (social‐evaluative threat) and whether he/she understands how to complete a task (out‐of‐control feeling). A key point is that although a child's perceived stress may not even constitute a valid stress from the teacher's viewpoint (Sotardi, 2016), it could potentially affect their receptivity to learning.

Yeager and Dweck (2012) proposed that subtle messages from adults influence pupils' belief systems – for example, adding the word ‘yet’ to what would be a negative sentence ‘You haven't done it*, yet*’ effectively diffuses the negativity by suggesting that the child will accomplish it at a later date (Haimovitz & Dweck, 2016). Dweck's ‘Growth Mind‐Set’ model proposes two mind‐sets that influence motivation and learning very differently. The limiting Fixed Mind‐Set assumes that abilities are fixed throughout life and cannot be changed by individual effort, and gives no recognition of personal growth: the failure‐is‐debilitating mindset. In contrast, the Growth Mind‐Set focuses more on the malleability of intelligence and so a difficult task is more likely to be viewed as a healthy challenge: the failure‐is‐enhancing mind set. Despite the plausibility of the model, nearly half the studies attempting to change mind‐sets which also verified the subsequent impact on mind‐set (some did not!) found that the manipulations did not produce a measurable change on pupils' mind‐sets (Sisk, Burgoyne, Sun, Butler, & Macnamara, 2018). However,   grade improvements through such manipulations have been noted for the lowest achieving students, so the intervention may work better for some children than others (Yeager et al., 2016, 2019). The apparent low malleability of mind‐sets may be because parents' mind‐sets influence children more strongly in this regard than those of teachers (Haimovitz & Dweck, 2016).

In the context of stress, Crum and colleagues proposed that two different mind‐sets, the ‘stress‐is‐enhancing’ mind‐set (embracing‐the‐challenge) versus the ‘stress‐is‐debilitating’ mind‐set (worrying‐about‐the‐challenge) can affect an individual's stress response (Crum, Salovey, & Achor, 2013). The stress‐is‐enhancing mindset may lead to more positive outcomes than the stress‐is‐debilitating mind‐set. This proposal can be tested by reappraisal interventions. Brooks (2014) defines reappraisal as 'a form of cognitive change that involves construing an emotion‐eliciting situation in such a way that changes its emotional impact' – for example, by using the simple self‐statement ‘*I am excited*’ to reappraise anxiety as the arousal‐congruent emotion of excitement (Brooks, 2014). There is some evidence that these types of reappraisal techniques can lead to short‐term changes – for example, university and high‐school students exhibited superior performance when a maths test was reframed as a challenge, rather than a threat (Brooks, 2014) (see also Crum, Jamieson, & Akinola, 2020) – but evidence for longer‐term effects is lacking. In addition, evidence that these types of reappraisal techniques can directly influence an individual's level of physiological stress responding has also not, to our knowledge, been shown.

#### Reappraising Learning

A more recent approach suggests a different route: that, for children from stressful environments, we should instead be reappraising how we present learning tasks to play to their strengths. This is along the lines recommended by Vogel and Schwabe (2016): it is important to understand pupils' inter‐individual differences to help develop more personalized training programs with a view to helping prevent pupils' stress–induced impairments. Children growing up in harsh environments may develop typical, or even enhanced, skills for solving problems in high‐adversity contexts (Ellis et al., 2020). Learning that is tailored to play to their strengths may be more effective. This approach may be considered as an extension of resilience science, the study of individuals exhibiting positive developmental outcomes despite exposure to adversities. In this way, the hidden talents model treats stress‐adapted skills, such as flexible switching between tasks or mental states or tracking novel information in the environment, as a form of adaptive intelligence that needs to be harnessed for individuals to achieve their full potential. The approach recommends restructuring school environments so that individuals with impaired sustained attention skills, who find it demanding to complete a lengthy task, are instead given tasks that utilize and build upon their hidden talents and strengths. At present, however, there are to our knowledge no intervention studies that have explicitly tested the effectiveness of this approach, and they would be predicated on being able to identify each child's individual profile of stress response.

#### Mindfulness

The notion of mindfulness introduced to the West, as used in Mindfulness‐Based Stress Reduction, was operationally defined by Kabat‐Zinn as ‘paying attention in a particular way: on purpose, in the present moment, and nonjudgmentally’ (Kabat‐Zinn, 1994, as reported by Purser & Milillo, 2016). Nowadays, a plethora of different versions of mindfulness have been developed, leading to criticism that the concept has been diluted (Purser & Milillo, 2016). An increasing number of schools have adopted mindfulness‐based interventions as a way of attempting to improve children's behavioral, cognitive and mental health outcomes. A key proposed role of mindfulness training is the reduction of stress.

The only meta‐analysis (*N* = 33 studies) to date of randomized controlled trials across a wide age group of children and adolescents (4‐year‐olds to 17‐year‐olds; Dunning et al., 2019) found support for small but significant effects of mindfulness‐based interventions, relative to all comparison conditions combined (including passive control groups), for the following outcomes: mindfulness, executive functioning, attention, depression, anxiety/stress and negative behaviors. Across the 17 randomized controlled trials that featured comparison to active control interventions (such as relaxation training), support for significant benefits of mindfulness‐based interventions was restricted to mindfulness, depression and anxiety/stress (Dunning et al., 2019). However, surprisingly, the outcomes were not significantly affected by the number of hours of training. Despite these positive findings, the meta‐analysis noted a lack of standardized interventions (22 different mindfulness protocols across the 33 studies) with many researchers developing and implementing their own personal mindfulness interventions instead of replicating results from previous studies using established mindfulness protocols. Publication bias was noted towards studies with positive results, and Dunning et al. suggested that further biases may have occurred from different methodologies and uncertainty over crucial design characteristics. They noted how the outcomes were typically measured by self‐report and recommended that future studies would be strengthened if observer‐rated measures were used along with direct physiological or behavioral measures; as mindfulness was developed to improve mental health, future studies should also include active control groups employing psychosocial and psychological interventions targeting the same outcome. Another meta‐analysis suggested that late adolescents (15‐year‐olds to 18‐year‐olds) may gain more benefits from mindfulness interventions than primary school children (Carsley, Khoury, & Heath, 2018). However, this study was not a randomized controlled trial and did not analyze effects on stress; moreover, Dunning et al.'s (2019) analysis yielded a mixed picture on age effects.

In sum, small but significant effects of reductions in depression and anxiety/stress have been found following mindfulness interventions with children and adolescents, potentially operating in part through reduction of stress, although there is currently a lack in the quantity and quality of evidence (Saunders & Kober, 2020).

### Physiological Factors

#### Breathing Techniques

Breathing, like other aspects of our autonomic nervous system, is under automatic control: we automatically start to breathe faster when we are doing exercise, or are anxious. But, unlike other aspects of our autonomic function (such as heart rate, or sweating), breathing is something that we can also voluntarily control when we want to. Because of this, and building on traditional techniques from the Eastern tradition such as pranayama, a number of authors have investigated whether slowing breathing by controlling the pace of in‐ and out‐breaths can alter physiological stress responses (Lehrer & Gevirtz, 2014; Noble & Hochman, 2019; Russo, Santarelli, & O'Rourke, 2017). Research on adults has established that nasal slow‐paced breathing (i.e., at around 6 breaths a minute compared with the typical 9–24 breaths per minute) can immediately alter stress‐related physiology, by shifting it toward increased activity within the parasympathetic nervous system – thus, for example, maximizing heart rate variability, associated with lower stress (Noble & Hochman, 2019). Optimization of several physiological variables has also been noted, for example maximizing oxygen absorption (through activating various receptors in the lungs) and beneficial cardiorespiratory effects (Russo et al., 2017). Breathing exercises have considerable practical potential: the advantage of using a specific breathing technique is that, once learned, it can be performed anywhere at any time and be easily included as an alternative ‘attention grabber’ for teachers to use.

However, research is still in its early stages, with only a few small exploratory studies conducted with adults and even fewer conducted with children. Heart rate variability and standardized anxiety scale measurements demonstrated significant improvements in a randomized controlled trial with a class of 8‐year‐olds after a 4‐month 10‐minute daily intervention that included deep breathing; the children reported that they were better able to cope with everyday stressors (Bothe, Grignon, & Olness, 2014). Heart rate variability measurements demonstrated improvements 1 year later. After a slow paced breathing intervention, a group of 14 adolescents with intellectual disabilities demonstrated improved stress management compared with the control group who had an audiobook session (Laborde, Allen, Göhring, & Dosseville, 2017). Khng (2017) used a deep breathing intervention just before a timed mathematics test in 122 10‐to‐11‐year‐old children, and reported that taking deep breaths before the test significantly reduced self‐reported feelings of anxiety and improved test performance compared to the control group. Nevertheless, compared to the research into mindfulness, there is an absence of randomized controlled trials looking at breathing interventions in adults as well as children. Even for adults, as Russo et al. (2017) point out, research into controlled, slow breathing should be extended into investigating long‐term effects rather than the short‐term effects that have been the focus of research to date.

In sum, although a few preliminary studies are suggesting that deep breathing may help primary school children to be more resilient when under stress, more robust research in the form of randomized controlled trials is required.

#### Recovery Time

The research reviewed in Part (c) suggests that stress can affect learning and memory over longer time scales, including impairing memory and learning minutes to hours after a stressful event. For children experiencing stressful home environments, breakfast clubs may help in delivering safe, nurturing school environments and nutritional support. In principle, they could also improve learning outcomes during the first two lessons for those vulnerable children who have experienced stress before arriving at school, by providing more time for delayed chemicals that suppress learning to dissipate, and therefore offer a buffer between home environment and learning activities. However, systematic evaluation of this possibility has yet to be carried out.

#### Physical Exercise

It has been proposed that physical exercise may benefit children's cognitive function by altering their stress‐related physiology (Álvarez‐Bueno et al., 2017; Wegner, Koutsandréou, Müller‐alcazar, Lautenbach, & Budde, 2019), with associated changes in activity levels of various signaling molecules in the brain (Strasser & Fuchs, 2015). In addition to direct effects of physical activity on stress responses (Puterman et al., 2018; Strasser & Fuchs, 2015), there are also various indirect routes through which physical activity is associated with reduced stress, such as exposure to fresh air, natural light, social interaction, and taking a break from work. Playing in team sports may also provide social support and reduce psychosocial stress (Keefe, Keefe, & Lavie, 2019). For example, a child struggling in a classroom may be a key member of social hierarchies within a school team, which potentially would reduce his or her psychosocial stress (Sapolsky, 2005).

However, whereas links between exercise and aspects of child development such as executive functions are well documented in meta‐analyses based on multiple randomized controlled trials (Chang, Labban, Gapin, & Etnier, 2012; Xue, Yang, & Huang, 2019), relatively fewer studies to date have examined the short‐ and long‐term effects of exercise on stress in children (Rasmussen & Laumann, 2013). Some correlational evidence has linked increased exercise with a reduced stress response in adults (Zschucke, Renneberg, Dimeo, Wüstenberg, & Ströhle, 2015) and exercise has been shown to reduce anxiety in children with Attention Deficit Hyperactivity Disorder (Cerrillo‐Urbina et al., 2015). More robust research, identifying cause‐and‐effect, is required to establish the most effective exercise for specifically reducing stress in primary school children.

### Environmental Factors

Noisy and chaotic environments adversely affect multiple aspects of child development (Marsh, Dobson, & Maddison, 2020). Increased stress is an important factor that may explain this relationship. For example, exposure to auditory noise is associated with physiological stress in children (Wass et al., 2019). Therefore, reducing noise and clutter within a learning environment (for example, through the use of noise‐dampening material to attenuate auditory noise) may be an effective stress management technique for some children.

While an environment can cause stress in children, it is also important to understand how stress can then influence how children interact with their environment. This is particularly important because as we have seen, elevated stress reduces children's ability to deploy focused attentional in a noisy, chaotic environment (Diamond et al., 2007; Liston et al., 2009). Reducing visual distractions and clutter within a classroom can help children maintain attentional focus and improve performance on learning tasks (Fisher, Godwin, & Seltman, 2014). It is likely – though it remains to be shown – that the challenge of maintaining attentional focus in a highly stimulating classroom would be greatest for children experiencing higher levels of stress (Diamond et al., 2007). However, because stress simultaneously increases vigilance, teachers can be aware that ‘attention grabbers’, such as clapping hands when children become distracted, should be more effective at eliciting attention in these same children.

## CONCLUSION

Some stress, manifested in changes to arousal and attention, is required for learning. Too much stress or stress at the wrong time may inhibit learning. This complex relationship, heuristically illustrated by an inverted‐U curve, is likely to differ between pupils depending on a multitude of long‐term and short‐term factors. Some children may be more sensitive to the environment than others. A classroom challenge providing optimal learning outcomes for one pupil may provide too much, or too little, arousal for another. Present‐day research as yet provides only a fragmentary framework for the development and testing of innovative top‐down psychotherapeutic and physiological interventions to regulate the physiological and neural stress response. We have described potential classroom strategies for addressing this issue, and explored the impact of environmental factors; however, at the current time, raising teachers' awareness of the inter‐individual differences in their pupils' stress responses will be an important step in accommodating the differing needs of children in their classrooms. Developing personalized approaches and training programs may prove to be the ultimate goal, to not only prevent stress‐induced impairments but also enable all children to achieve their full potential.
